# Azine- and Azole-Functionalized Oligo´ and Polythiophene Semiconductors for Organic Thin-Film Transistors

**DOI:** 10.3390/ma3031533

**Published:** 2010-03-03

**Authors:** Rocío Ponce Ortiz, He Yan, Antonio Facchetti, Tobin J. Marks

**Affiliations:** 1 Department of Chemistry, Northwestern University, 2145 Sheridan Road, Evanston, Illinois, 60208, USA; E-Mail: a-facchetti@northwestern.edu (A.F.); 2 Polyera Corporation, 8045 Lamon Avenue, Skokie, Illinois, 60077, USA; E-Mail: hyan@polyera.com (H.Y.)

**Keywords:** azine, azole, thiophene, organic field-effect transistors

## Abstract

In the organic electronics research field, several strategies have been used to modulate the transport properties of thiophene-derived semiconductors via sequential functionalization of their π-conjugated cores. This review summarizes the major design and synthetic strategies for tuning thiophene-containing small molecule and polymer properties by introducing electron-deficient nitrogen-containing azine and azole moieties. Several examples are presented which elucidate the structural, optical, and electronic consequences of incorporating these electron-deficient fragments in the conjugated skeletons, particularly relating to applications in organic thin-film transistors.

## 1. Introduction

### 1.1. General Overview

Over the last few years, thiophene-based small molecules and polymeric semiconductors [1] have attracted increasing attention for applications in diverse opto-electronic devices, such as organic field-effect transistors (OFETs) [2,3,4,5,6], light-emitting diodes [7,8], lasers [9], sensors [10,11], and photovoltaic cells [12]. Some of the principal attractions of oligo/polythiophenes include their remarkable chemical/electrochemical stability, broad accessibility of diverse thiophene synthons, efficient tunability of their electronic properties by substitution at terminal and internal skeletal positions, and the well-developed/regioselective ring-ring coupling methodologies available [13,14]. Because of the electron-rich nature of the thiophene ring, unsubstituted and alkyl-substituted oligo/polythiophenes are p-channel (hole-carrying) semiconductors in organic field-effect transistors (OFETs) [15,16,17]. Note however that one drawback of these systems is the relatively high energy of the highest occupied molecular orbital (HOMO), which usually leads to air-sensitivity and oxidatively doped “always-on” OFET devices [18]. In this sense, the results of recent studies [19,20] suggest that a modest reduction of the HOMO energy, achievable by functionalization with electron-withdrawing groups or introduction of electron-poor moieties/rings, should yield materials less susceptible to air oxidation. Furthermore, since HOMO energy reduction is usually accompanied by a lowering of the LUMO level as well, n-channel (majority electron-transporting) or ambipolar transport (both hole and electron transporting) materials should be possible. Note that both p- and n-channel transistors are required for fabricating complementary (CMOS) circuits.

Since the first demonstration of p-channel FET operation in oligothiophenes [21,22,23,24], a variety of synthetic strategies have been used to modify oligothiophene chemical and electronic structure, with the aim of enhancing air stability and/or inverting the polarity of the majority of charge carrier from p- to n-channel. Regarding achieving n-channel transport, the most successful strategies involve the introduction of fluorocarbon- [25,26], fluoroarene-, alkyl/fluoroalkycarbonyl-substituents as well as cyano groups [27,28,29] on the conjugated skeleton. Device performance has been optimized by perfecting film growth processes using selected solvents, deposition temperatures, solvent or thermal annealing processes, and/or pairing with semiconductor properties-enhancing gate dielectrics [30,31,32]. Another strategy, which will be discussed in detail in this review, includes oligothiophene core functionalization with nitrogen-containing, electron-poor heterocycles. Following this approach a significant number of small molecules [33] and polymers have been synthesized [34,35,36,37]. Interestingly, even if azine functionalization substantially lowers the orbital energies of these systems, n-channel or ambipolar transport is not always achieved [38], although air stability is improved significantly. Thus, electron transport has been observed in azine/azole-thiophene derivatives when other electron-withdrawing groups are introduced [39]. 

Recent experimental and theoretical results have demonstrated that oligothiophene molecular electronic structure and FET majority charge transport properties respond in very different, and not necessarily intuitive ways to semiconductor skeletal modifications [40]. These observations highlight the importance of analyzing the results obtained so far in order to better understand the structural and electronic effects of introducing azine and/or azole rings in thiophene-containing small molecules and polymers. To this end, the electronic structure and the electrical performance of azine- and azole-substituted oligo- and polythiophene semiconductors will be discussed.

### 1.2. Organic Field-Effect Transistor Structure and Operation

**Figure 1:**
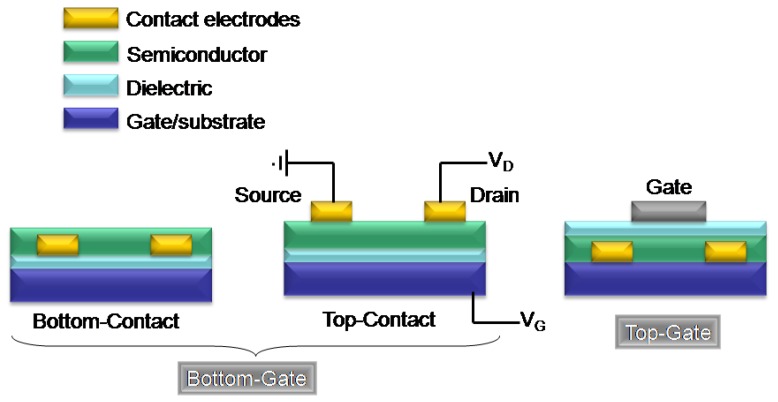
Various configurations of organic field-effect transistors.

Figure 1 shows the typical configurations used for OFETs [41,42,43], which are composed of three fundamental elements, contacts (source, drain, and gate), the semiconductor, and the dielectric layers. The arrangement of these components can be classified as either bottom-gate or top-gate, and there are three common device geometries (Figure 1).

In the FET examples presented here, the bottom-gate structure is that most commonly used, and both top-contact, and bottom contact configurations are discussed. The electrical performance is estimated in terms of standard transistor performance characteristics, where the field-effect mobility (*μ*) can be extracted from the linear regime (Equation 1) or from saturation regime (Equation 2) I-V data [44].

(1) $${\left(I_{SD}\right)}_{lin}=(W/L)\mu C(V_{G}-V_{T}-V_{D}/2)V_{D}$$

(2) $${\left(I_{SD}\right)}_{sat}=(W/2L)\mu C{\left(V_{G}-V_{T}\right)}^{2}$$

In Equations 1 and 2, *(I_SD_)_lin_* is the drain current in the linear regime, *(I_SD_)_sat_* is the drain current in the saturation regime, *μ* is the field-effect carrier mobility of the semiconductor, *W* the channel width, *L* the channel length, *C* the capacitance per unit area of the gate insulator layer, *V_T_* the threshold voltage, *V_D_* the drain voltage, and *V_G_* the gate voltage. On increasing *V_D_* and *V_G_*, a linear current regime is initially observed at low drain voltages (*V_D_* < *V_G_*) (Equation 1), followed by a saturation regime when the drain voltage surpasses the gate voltage (Equation 2).

## 2. Azine/Azole-Thiophene Oligomers

### 2.1. P-Channel Semiconductors

**Figure 2:**
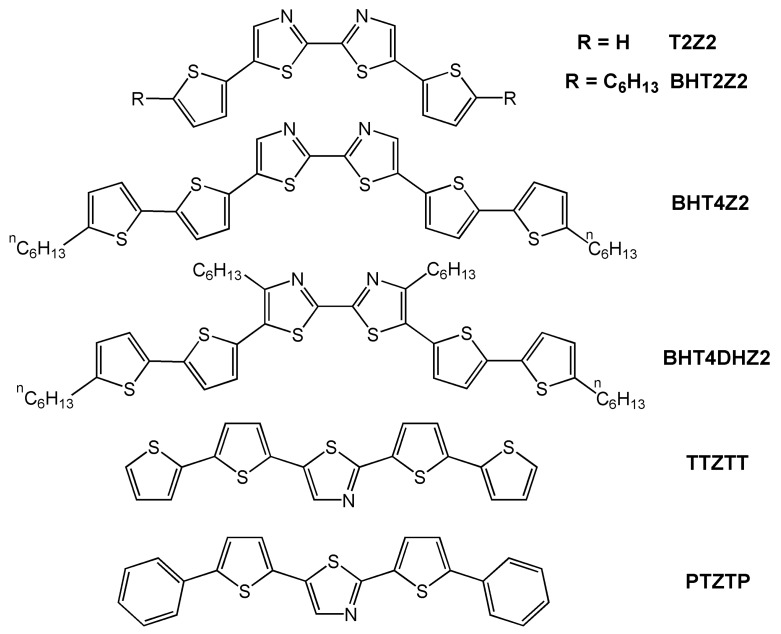
Chemical structures of thiazole-containing oligomers.

Table 1 summarizes the properties of the various azine- and azole-derived oligothiophenes presented in this section of the review. The first example of azole- containing oligothiophenes for OFETs was reported by Katz *et al.* in two different contributions. In the first [20], the authors analyzed systems analogous to alkyl-substituted tetrathiophenes and sexithiophenes where the two internal rings are thiazole groups (see **T2Z2**, **BHT2Z2**, **BHT4Z2** and **BHT4DHZ2** in Figure 2). These molecules were designed to lower the HOMO level with respect of the corresponding oligothiophenes, thus making them less susceptible to air-promoted p-doping.

Katz *et al.* [20] found that incorporation of thiazole rings into oligothiophenes has a profound effect on the properties of the vapor-deposited thin films. The authors argued that the greater polarity introduced by the azine nitrogens influences the crystal growth, probably decreasing the lateral interactions between the π-systems, which is translated into lower mobilities. Furthermore, less efficient charge injection is expected in these systems due to its decreased electron-richness and HOMO energies, resulting in a greater energy mismatch between the work function of the Au source/drain electrodes and the molecular HOMO level energies. **BHT4Z2**–based FETs exhibit the largest field-effect mobility of 0.0077 and 0.011 cm^2^V^-1^s^-1^ for films vapor- deposited at 30 °C and 55 °C, respectively. Lower mobilities by at least one order of magnitude are found for the other thiazole-containing oligomers. These results were explained in terms of the molecular packing in the solid-state structures as well as the film morphologies. In fact, **BHT4Z2** films exhibit a molecular growth orientation similar to that of α,ω-dialkylsexithiophenes [45,46], while the compounds **T2Z2** and **BHT2Z2** exhibit two unrelated families of film x-ray diffraction Bragg progressions, suggesting two different crystallite orientations. Furthermore, morphology studies on films of these two molecules reveal crystallite orientations less favorable for charge transport compared to films of **BHT4Z2**. Note that despite the lower mobilities of these thiazole oligomers compared to the analogous alkyl-oligothiophenes, these films exhibit far greater air stability due to lower HOMO energies. Current on/off ratios greater than 10^4^ in air are routinely achieved as well as negative threshold voltages that are essentially invariant with time in air.

In Katz´s second paper [47] a different strategy is reported in which only a single thiazole ring was introduced in the center of the oligothiophene backbone (**TTZTT** in Figure 2), and the effect of phenyl group introduction was also analyzed (**PTZTP** in Figure 2). Electronic structure calculations predict a HOMO orbital stabilization from −4.86 eV in pentathiophene to −5.05 eV in **TTZTT**, and a further stabilization to −5.13 eV in **PTZTP**. The authors found that for films grown by vacuum evaporation at room temperature, the mobilities are lower in the thiazole-thiophene compounds (6 × 10^-5^ cm^2^V^-1^s^-1^ for **TTZTT** and 10^-4^ cm^2^V^-1^s^-1^ for **PTZTP**) *versus* pentathiophene (5.7 × 10^-3^ cm^2^V^-1^s^-1^), however the mobility of **TTZTT** can be increased to 7 × 10^-3^ cm^2^V^-1^s^-1^ for films deposited at 60–80 °C. The authors also found that in general the crystallites of thiazole-containing films are smaller and more disordered than those of homologous systems without azine rings, which together with the depressed HOMO, can explain the lower mobility. In contrast, XRD analysis of these films reveals nearly perpendicular molecular orientations for both the pentathiophene and thiazole-oligothiophene long axes *versus* the substrate surface, thus ruling out molecular misaligment as a source of the reduced mobility.

**Figure 3:**
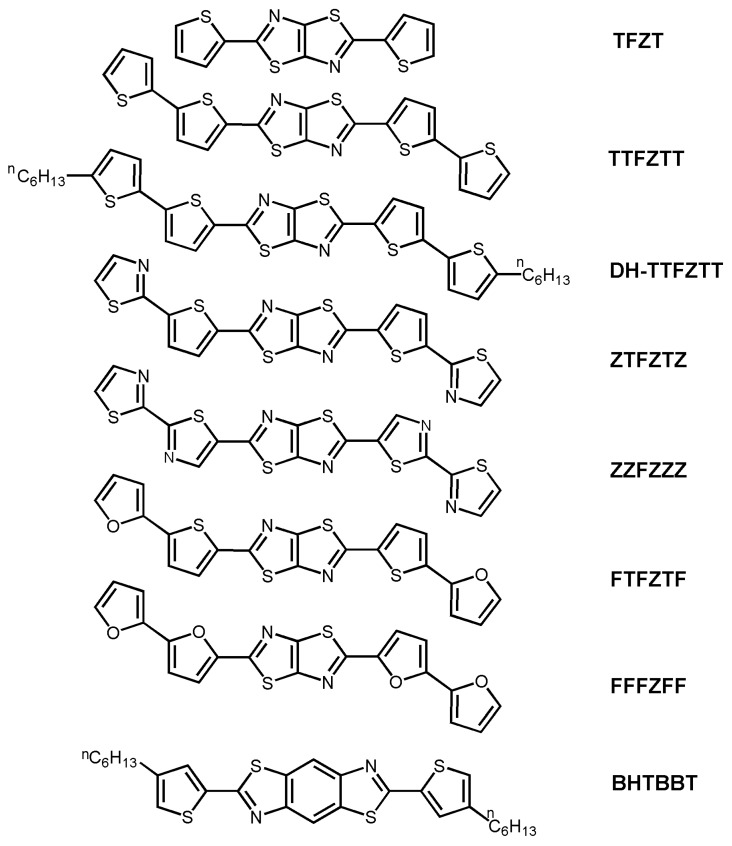
Chemical structures of thiazolothiazole oligomers and benzobisthiazole **BHTBBT**.

**Figure 4:**
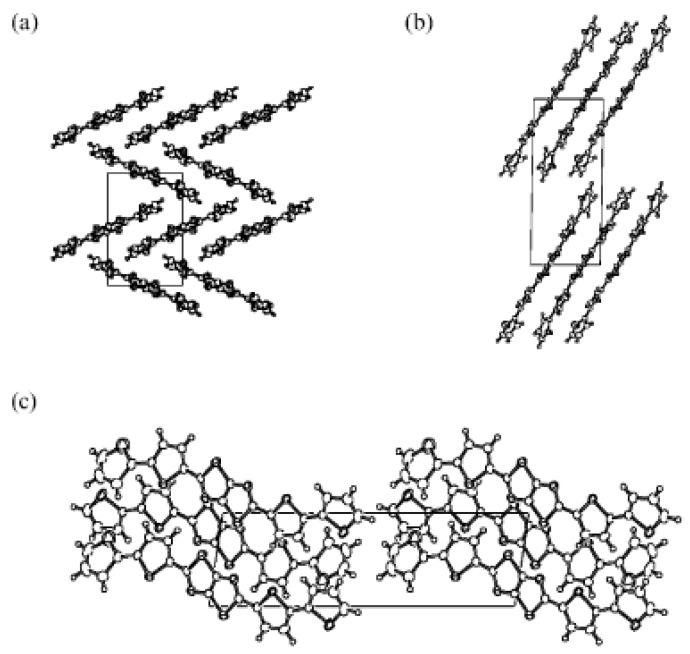
Packing view along the *a*-axis of **TFZT** (a), **TTFZTT** (b), and the *b*-axis of **TTFZTT** (c). From reference [48], DOI: 10.1039/b403699a. Reproduced by permission of the Royal Society of Chemistry.

A significant increase in the electrical performance was reported by Yamashita *et al.* [48,49] by using thiophene/thiazolothiazole co-oligomers instead of thiophene/thiazole co-oligomers. The thiazolothiazole building block, as well as thiazole, is an electron-poor unit leading to donor-acceptor compounds when combined with thiophene. The resulting structures exhibit lower HOMO-LUMO gaps, higher carrier mobilities, and even ambipolarity [27], as well as increased stability to ambient atmosphere. An additional attraction *versus* thiazole-containg systems is the rigid structure of the thiazolothiazole unit due to ring fusion. This rigidity may lead to reduced reorganization energies as well as considerable π-π intermolecular overlap, which should enhance charge transport. In a first report [48], the authors synthesized thiophene-thiazolothiazole derivatives (see chemical structures of **TFZT**, **TTFZTT** and **DH-TTFZTT** in Figure 3) and found efficient solid state π-π stacking for **TTFZTT** (see Figure 4). Devices based on this system exhibit a field-effect mobility of 5 × 10^-3^ cm^2^V^-1^s^-1^, threshold voltage of −20 V, and on/off ratio of 10^3^, when the thin-films were deposited at 20 °C, and 2 × 10^-2^ cm^2^V^-1^s^-1^, −7 V, and 10^4^, when deposited at 50 °C. AFM images indicated larger crystalline grains and smoother surfaces at higher growth temperatures. Introduction of n-hexyl groups, **DH-TTFZTT**, decreased the threshold voltage to −2 V while maintaining the ~10^-3^ cm^2^V^-1^s^-1^ hole mobility for room temperature growth, probably due to the highly ordered structure caused by some degree of intermolecular interdigitation of the alkyl groups. In contrast, **TFZT** FETs are inactive probably due to the combination of the small core and high ionization potential of this molecule (it presents an oxidation potential of 1.43 V *vs.* 1.12 V and 1.02 V for **TTFZTT** and **DHTTFZTT**, respectively, *vs.* SCE). This compound exhibits a herringbone type crystal structure (Figure 4).

In a following contribution, Yamashita *et al.* [49] modified **TTFZTT** by replacing the thiophene rings with other five-membered heterocycles such as furan and thiazole (see chemical structures in Figure 3). However, this modification was not successful since while the electrochemically-derived HOMO levels of the furan derivatives are higher than that of **TTFZTT,** lower FET performance is achieved. Note that electrochemical experiments indicated minimal electron transfer ability for **ZZFZZZ** since it exhibited no oxidation wave and only an irreversible reduction wave at −0.92 V *vs.* SCE. **ZTFZTZ**- and **ZZFZZZ**-based FETs are inactive which was attributed to, among other factors, non-optimal molecular orientations within the thin films.

Following the same strategy of a fused unit, Skabara *et al.* [50] synthesized in 2007 a new molecule, bis(hexylthieno)benzobisthiazole (**BHTBBT**), which self-assembles in the solid state to give intermolecular close contacts in the three cartesian directions. The unique π-stacking that this molecule presents together with strong intermolecular interactions yields a hole mobility as high as 10^-2^ cm^2^V^-1^s^-1^, threshold voltage of approximately −20 V and on/off ratio of 10^5^. It is also interesting to note that using theoretical calculations the authors stated that this material should be expected to show 3D conduction properties, but presenting anisotropy in at least one direction. 

**Figure 5:**
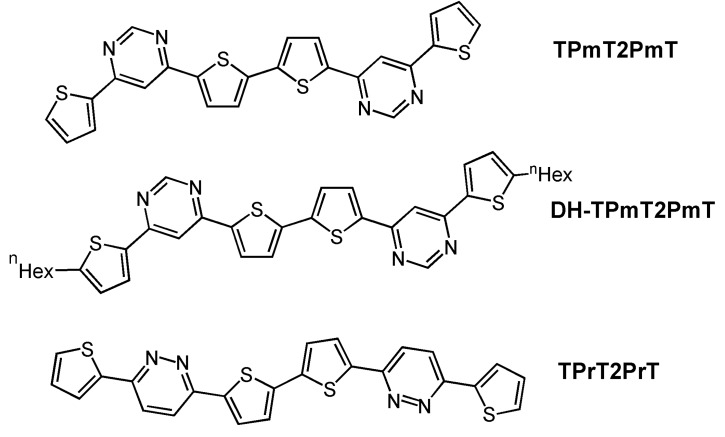
Chemical structures of thiophene-diazine oligomers.

Quite recently, some of us have reported a new family of mixed heteroarene-oligothiophene semiconductors [40] (see Figure 5). In this case, these nitrogen-containing heterocycles (pyrimidine and pyridazine groups) were selected because of their more positive reduction potentials and greater electron-withdrawing capacities than other monocyclic pyridines and thiazoles [51]. In fact, both oxidation (irreversible processes) and reduction waves are observed by cyclic voltammetry for the three oligomers, indicating the possibility of ambipolarity in FET device performance. The electrochemical gap is larger in the three molecules (3.05 eV for **TPmT2PmT**, 3.28 eV for **TPrT2PrT** and 3.08 eV for **DH-TPmT2PmT**) than in sexithiophene (2.69 eV), and the optical gaps of the diazine-thiophene oligomers indicate an electronic structure in-between those of bithiophene and tetrathiophene. Electrical characterization of the corresponding devices indicated that all of the new materials exhibited only p-channel transport. Interestingly, electron transport is not observed although the diazine-thiophene semiconductors are clearly more electron-deficient than their p-channel oligothiophene analogues. The highest mobility was recorded for **TPrT2PrT**, with a value of 4 × 10^-3^ cm^2^V^-1^s^-1^, threshold voltage of −68 V, and on/off ratio of 10^7^. This greater hole mobility *vs.* **TPmT2PmT** (1 × 10^-4^ cm^2^V^-1^s^-1^) is likely a result of a more linear geometry in the case of **TPrT2PrT**, as shown by theoretical calculations, due to *para* connections between the thiophene and pyridazine fragments as opposed to *meta* connections found in thiophene-pyrimidines. In the latter, the *meta* functionalization allows two different conformations which may create packing defects and reduce charge transport efficiency.

In order to explain the absence of electron transport in these molecules, their reorganization energies were estimated using DFT computation. The results indicated that, with the exception of **TPrT2PrT**, reorganization energies for anions, which are the molecular parameter relevant to electron transport, are larger than those predicted for cations. Futhermore, the n-hexyl substitution in **DH-TPmT2PmT** decreases the hole reorganization energy by 0.03 eV compared to that of **TPmT2PmT**, in agreement with the increased p-channel mobility found in the electrical characterization (3 × 10^-3^ cm^2^V^-1^s^-1^ for **DH-TPmT2PmT** *vs.* 1 × 10^-4^ cm^2^V^-1^s^-1^ for **TPmT2PmT**). Finally, it was argued that the electrical behavior of these new semiconductors could be explained considering that thiophene-diazine properties are dominated by the central bithiophene fragment. In this sense, the diazine-thiophene semiconductors behave as conventional hole-semiconducting oligothiophenes with properties similar to those of medium-sized oligothiophenes, as confirmed in their optical properties (optical band gaps).

**Table 1:** Summary of the electrical performance (mobility, μ, current on/off ratio, I_on_/I_off_ and threshold voltage, V_T_) of the of various azine- and azole-derived oligomers. Top-contact device architectures are utilized unless otherwise specified.

Oligomer	Dielectric^a^	T_D_ (°C)	μ_h_ (cm^2^V^-1^s^-1^)	V_T_ (V)	I_on_/I_off_	Ref.
**T2Z2^b^**	S	25	1 × 10^-5^	NR	NR	20
**BHT2Z2^b^**	S	25	2 × 10^-5^	NR	NR	20
**BHT4Z2**	S	55	0.011	−11	>10^4^	20
**BHT4DHZ2**	S	25	3.5 × 10^-4^	NR	NR	20
**TTZTT**	S	60–80	7 × 10^-3^	−7	10^4^	47
**PTZTP**	S	25	1 × 10^-4^	−23	80	47
**TTFZTT**	S	50	2 × 10^-2^	−7	10^4^	48
**DH-TTFZTT**	S	20	3 × 10^-3^	−2	10^3^	48
**ZTFZTZ^b^**	S	20	1 × 10^-7^	NR	10^3^	49
**FTFZTF**	S	70	1 × 10^-3^	NR	10^2^	49
**FFFZFF**	S	50	4 × 10^-4^	NR	10^3^	49
**BHTBBT^b^**	H	25	0.01	−20	10^5^	50
**TPmT2PmT**	H	70	1 × 10^-4^	−88	2 × 10^5^	40
**DH-TPmT2PmT**	H	90	3 × 10^-3^	−55	2 × 10^7^	40
**TPrT2PrT**	H	110	4 × 10^-3^	−68	10^7^	40

^a^ S: untreated Si/SiO_2_ gate dielectric; H: HMDS-functionalized Si/SiO_2_ gate dielectric. ^b^ Bottom-contact devices. NR: Not reported.

### 2.2. N-Channel Semiconductors

The previous section highlights the difficulty in achieving electron transport solely by the introduction of azine and azole rings in oligothiophenes, indicating the need for additional functionalization with stronger electron-withdrawing groups. The strategy that will be discussed in this section is the use of the trifluoromethylphenyl group in functionalizing π-conjugated systems, which has been extensively investigated by Yamashita *et al.* [39]. Table 2 summarizes the properties of the various n-channel azine- and azole-containing oligothiophenes presented in this section.

**Figure 6:**
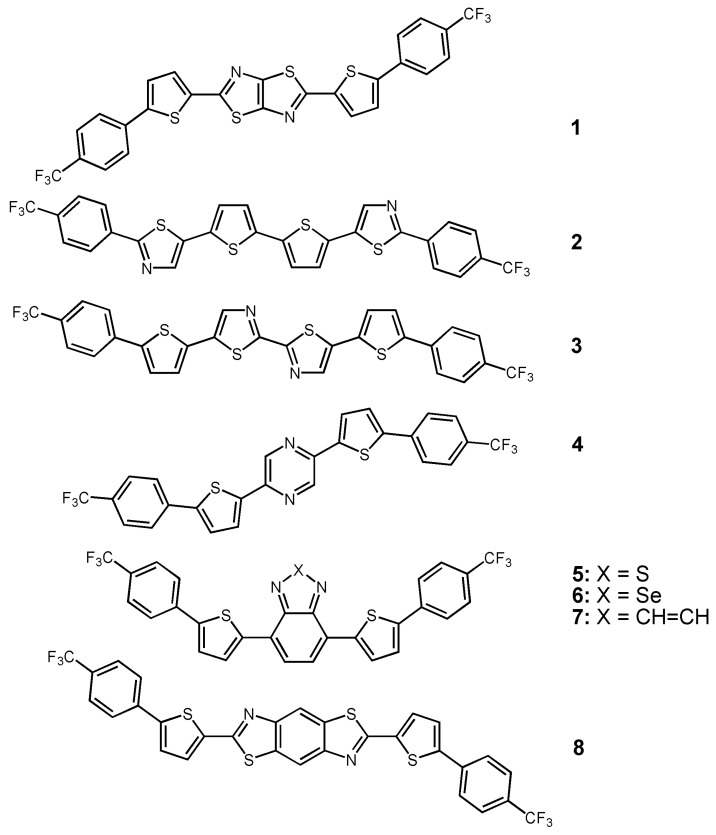
Chemical structures of trifluoromethylphenyl-functionalized n-channel azine- and azole- oligothiophenes.

The first example of electron transport in this type of azine- or azole- derived oligothiophene was reported in 2005 [52]. In this case, a thiazolothiazole group was introduced in-between two thiophene rings having trifluoromethylphenyl substituents (see chemical structure of **1** in Figure 6). The electron mobilities of **1** calculated in the saturation regime varied with the film deposition temperature, and reached ~0.30 cm^2^V^-1^s^-1^, threshold voltage of 60 V, and on/off ratio of 10^6^, for a deposition temperature of 50 °C on untreated Si/SiO_2_ substrates. The mobility was increased up to 1.20 cm^2^V^-1^s^-1^ for OTS-modified SiO_2_ substrates [53]. The π-stacking structure and the formation of a columnar motif in the single crystal of **1** (with short S^∙∙∙^S heteroatom contacts of 3.25 Å) may explain the high FET performance. Furthermore, thin-film XRD patterns indicate that molecules of **1** are almost perpendicular to the substrate surface, this being a favorable orientation for efficient charge transport. This example indicates that, as in the previous section, the thiazolothiazole unit favors the formation of stacked structures *versus* herringbone structures in oligothiophenes, and the trifluoromethylphenyl group is a very effective group to induce FET electron transport.

In a following communication [54], the authors combined thiophene and thiazole rings with the trifluoromethylphenyl substituents (see the chemical structures of **2** and **3** in Figure 6), and again succeeded in obtaining electron transport. In this case, the electron mobilities were lower in **2** and **3** compared to **1**, due to the loss of core rigidity by the replacement of the thiazolothiazole by non-fused thiazole groups. Nevertheless, electron mobilities of 0.085 and 0.018 cm^2^V^-1^s^-1^ were reported for **2** and **3**, respectively. The comparisons of FET performance of **2** with bis(4-trifluoromethylphenyl)tetrathiophene indicate that the electron transport is promoted by synergistic thiazole incorporation and trifluoromethylphenyl substitution of the oligothiophene core.

In order to increase the electron affinity in this series, the two thiazole rings of **3** were substituted by a pyrazine group (see the chemical structure of **4** in Figure 6) [55]. A field-effect mobility of 8.3 × 10^-4^ cm^2^V^-1^s^-1^ was obtained for semiconductor **4** in a bottom-contact geometry for films deposited at 50 °C. The authors also found that substitution of the thiophene rings of **4** for thiazole rings increased the FET performance; an electron mobility of ~0.04 cm^2^V^-1^s^-1^ for top contact devices was reported. Interestingly, but not unexpectedly, the substitution of the trifluoromethylphenyl groups in **4** for thiophene results in p-channel transport, indicating that the –C_6_H_4_CF_3_ is largely responsible for n-channel operation. 

Another strategy used by Yamashita *et al.* [56] was the substitution of the pyrazine group by a benzothiadiazole (**5**), benzoselenadiazole (**6**), and quinoxaline group (**7**). The best FET performance was found for compound **5**, which exhibits an electron mobility of 0.19 cm^2^V^-1^s^-1^ and a very low threshold voltage of 3 V for a top-contact device structure. This result can be ascribed to the combination of both electron-withdrawing benzothiadiazole and trifluoromethylphenyl groups. The substitution of the central sulfur thiophene for selenium (**6**) gives similar electrical performance in bottom-contact devices. In contrast, the quinoxaline derivative **7** exhibits the lowest electron mobility in this series, probably due to the herringbone packing structure found in the single crystal X-ray analysis *versus* the columnar structure found in single crystals of compound **6**.

Finally, as an extension of the previous studies [52] on thiazolothiazole-based oligomer **1**, Yamashita *et al.* focused on the benzo[1,2-*d*:4,5-*d*´]bisthiazole (BBT) ring, which is a π-extended derivative of thiazolothiazole [57]. Using this new approach they synthesized the semiconductor **8** (Figure 6) with the aim of increasing π-conjugation and intermolecular interactions, as shown in the previous section. A field-effect mobility of 0.24 cm^2^V^-1^s^-1^ was recorded for **8** (compared to 1.20 cm^2^V^-1^s^-1^ measured for **1**), which is due to the different solid-state intermolecular interactions. In fact, a herringbone crystal packing was found in **8** *vs.* the π-π stacking of **1**. Nevertheless, the introduction of BBT unit was successful in decreasing the threshold voltage, probably due to a better contact between the semiconductor and the electrodes in the herringbone structure. 

**Table 2:** Summary of the electrical performance (mobility, μ, current on/off ratio, I_on_/I_off_, and threshold voltage, V_T_) of n-channel azine- and azole-derived oligomers. Top-contact device architectures are utilized unless otherwise specified.

Oligomer	Dielectric^a^	T_D_(°C)	μ_e_ (cm^2^V^-1^s^-1^)	V_T_ (V)	I_on_/I_off_	Ref.
**1**	O	25	1.2	67	10^7^	53
**2**	S	25	0.085	63	10^4^	54
**3**	S	25	0.018	61	10^4^	54
**4^b^**	S	50	8.3 × 10^-4^	105	2.8 × 10^6^	55
**5**	H	80	0.19	3	10^6^	56
**8**	O	50	0.24	24	3 × 10^6^	57

^a^ S: untreated Si/SiO_2_ gate dielectric; H: HMDS-functionalized Si/SiO_2_ gate dielectric; O:OTS-functionalized Si/SiO_2_ gate dielectric. ^b^ Bottom-contact devices.

## 3. Azine- and Azole-Containing Polymers

**Figure 7:**
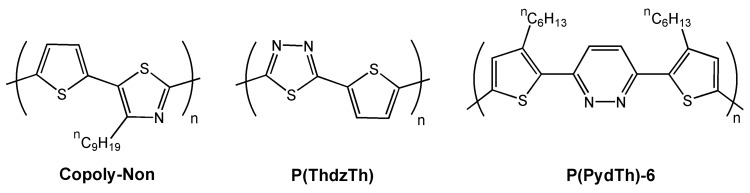
Chemical structures of some copolymers for field-effect transistors.

One of the first examples of field-effect transistors fabricated with azole- and azine-containing copolymers was reported by Yamamoto *et al.* in 2004 [58]. They synthesized donor-acceptor type copolymers of thiophene and 4-alkylthiazole, and analyzed the electrical behavior of the corresponding regioregular polymer with an n-nonyl alkyl chain (see chemical structure of **Copoly-Non** in Figure 7). The authors ascribed the quite high hole mobility of **Copoly-Non** (~2.5 × 10^-3^ cm^2^V^-1^s^-1^) to the alignment of the polymer on the HMDS-treated surface with the alkyl groups oriented toward the dielectric surface. A year later, in an attempt to increase the electron-affinity, they reported a new charge transfer copolymer (**P(ThdzTh)** in Figure 7) using a greater electron-withdrawing heterocycle (1,3,4-thiadiazole) [38].

**Figure 8:**
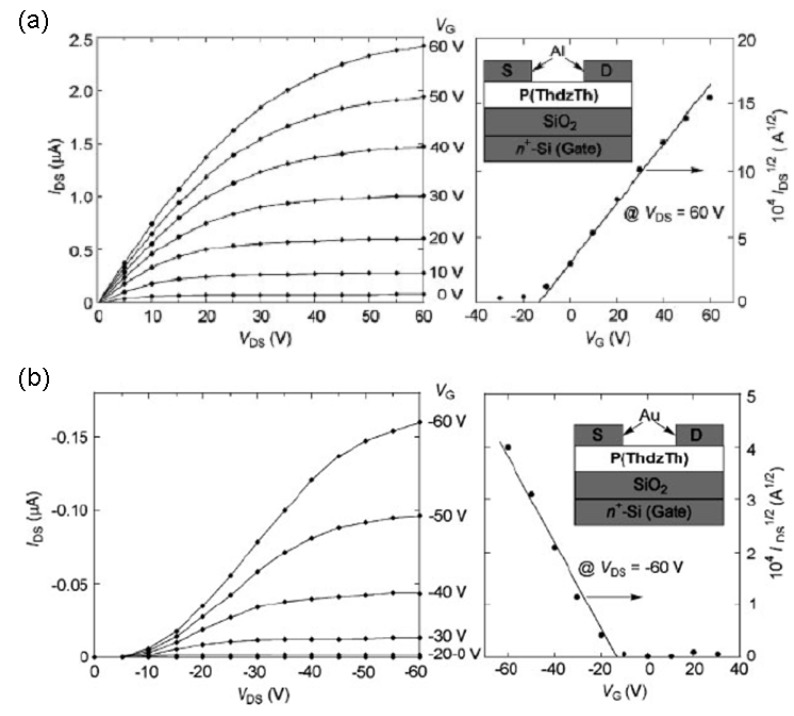
Output and transfer characteristics of **P(ThdzTh)**-based FETs for n-channel operation (a), and for p-channel operation (b). The insets in (a) and (b) show the structures of the corresponding top-contact FET devices. Reproduced with permission from reference [38]. Copyright 2005 Wiley-VCH Verlag GmbH & Co. KGaA.

This strategy was successful since they achieved a copolymer exhibiting facile electrochemical p- and n- doping and also ambipolar FET transport. The authors fabricated top-contact FET devices by spin-coating **P(ThdzTh)** onto hexamethyldisilazane (HMDS)-treated substrates and using different electrodes to enable p-channel (Au) or n-channel (Al) field-effect transistors. Figure 8 shows the electrical performance of the corresponding devices, where both electron and hole transport, with metal similar carrier mobilities of 5.4 × 10^-3^ cm^2^V^-1^s^-1^ and 3.4 × 10^-4^ cm^2^V^-1^s^-1^, respectively, is realized. The authors attributed the respectable mobilities to the ordered face-to-face π-stacked solid structure of the copolymer, with an intermolecular π-π distance of approximately 3.7 Å [59], and the good electron mobility to the better energy matching of the LUMO (−3.4 eV) of **P(ThdzTh)** to the workfunction [60] of Al (4.0 eV) *vs.* Au (5.1 eV) electrodes.

**Figure 9:**
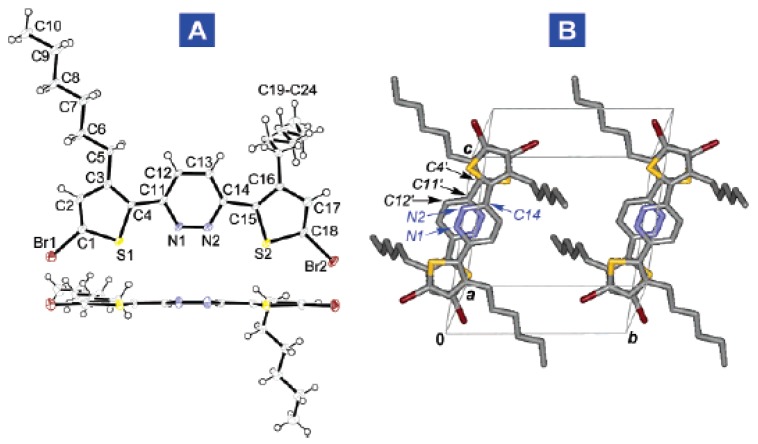
(A) ORTEP drawing and (B) crystal packing diagram of the monomer building block of **P(PydTh)-6** copolymer. Reproduced with permission from reference [36]. Copyright 2005 American Chemical Society.

Another strategy reported by Yamamoto *et al.* [36] was to alternate electron-donating thiophene or 3-alkylthiophene groups with the electron-poor pyridazine rings (see chemical structure of **P(PydTh)-6** in Figure 7). Pyridazine is one of the most electron-poor azaheterocycles because of the presence of two azine nitrogen atoms. Another advantage that these thiophene-pyridazine copolymers offer is the presence of S^∙∙∙^N interactions [61,62,63] that may direct molecular self-assembly in the solid state. In fact, in order to analyze the crystal packing, the authors resolved the crystal structure of the monomeric building block (see Figure 9) and found that the three aromatic rings are highly coplanar, indicating that the steric repulsions between the thiophene-alkyl groups and the hydrogen atoms of the pyridazine ring are negligible. This could be due to the aforementioned S^∙∙∙^N intramolecular nonbonded interactions, which force the thiophene rings to bend toward the pyridazine nitrogen. Furthermore, the crystal packing of this monomer (Figure 9B) consists of infinite molecular π-stacked columns along the *a* lattice direction. In these pillars, the molecules are arranged in an antiparallel fashion, reflecting the orientation of the central pyridazine units, resulting from dipole-dipole interactions. 

This packing motif features strong intermolecular interactions between the stacked molecules (interfacial packing distance of 3.4 Å), which is desirable for charge transport. In regard to the polymer microstructure, XRD patterns of the films reveal coplanar π-stacked chains in the solid state, with the face-to-face π-stacked distance almost identical (3.5 Å) as that in the monomer. Interdigitation of the alkyl groups has also been proposed for this polymer in the solid state.

Thin-film field-effect transistors based on **P(PdyTh)-6** exhibit only p-channel characteristics, with a hole mobility of 3 × 10^-3^ cm^2^V^-1^s^-1^ and a current on/off ratio of 4 × 10^3^. In this case, compared with the previous work on **P(ThdzTh)**, the absence of electron transport can be ascribed to the large barrier for electron injection from the Au electrodes (used in the **P(PdyTh)-6**-based devices) into the polymer and to the slightly destabilized LUMO of **P(PdyTh)-6** (−3.0 eV) compared to that of **P(ThdzTh)** (−3.4 eV).

**Figure 10:**
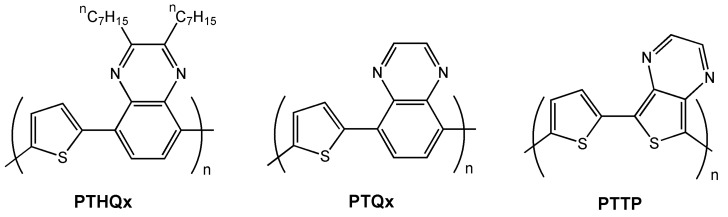
Chemical structures of the semiconducting polymer **PTHQx** and theoretical model structures **PTQx** and **PTTP**.

Jenekhe *et al.* [64] synthesized the new thiophene-quinoxaline donor-acceptor conjugated copolymer **PTHQx** (Figure 10). The authors also investigated computationally the electronic structures and properties of the related polymers **PTQx** and **PTTP** (Figure 10) with the aim of understanding the effect of intramolecular charge transfer on the polymer properties and charge transport in FET devices. XRD analysis of **PTHQx** thin films reveals a high degree of crystallinity while AFM images indicate a polycrystaline grain morphology similar of that commonly observed for thermally evaporated small molecule semiconductors such as pentacene [65]. Bottom-contact devices were fabricating using untreated and octyltrichlorosilane-treated Si/SiO_2_ substrates as gate/dielectrics (OTS-8), and a saturation hole mobility of 2.7 × 10^-5^ cm^2^V^-1^s^-1^ with an on/off ratio of 10^3^ was reported with an unmodified SiO_2_ dielectric and film annealed at 105 °C for 10 min, and 2.6 × 10^-4^ cm^2^V^-1^s^-1^ and on/off ratio of 10^4^ for films annealed at 150 °C for 10 min. Surface modification of the dielectric layer with an OTS-8 monolayer led to significant improvement in FET performance, with a field effect mobility of 3.6 × 10^-3^ cm^2^V^-1^s^-1^, on/off ratio of 6 × 10^5^, and threshold voltage of −2 V when the devices where annealed at 60 °C overnight. These results indicate that dielectric surface treatment, film annealing temperature, and annealing time are important parameters that must be controlled to optimize polymer-based OFET performance.

Theoretical calculations on the model compound **PTQx** indicate that the thiophene and quinoxaline units are not coplanar in the copolymer, which may impede optimized chain packing. In contrast, the theoretical model **PTTP** contains a coplanar structure and a quinoid geometry (aromatic for **PTQx**), indicative of a large degree of intramolecular charge transfer. Also related to the planarity of its structure, **PTTP** has a lower theoretical band gap (1.04 eV) than **PTQx** (2.18 eV). These theoretical results suggest that **PTTP**–based polymers should be better charge-carrying materials. Nonetheless, the computed HOMO energies of the two copolymers suggest facile injection of holes for both systems. In contrast, the computed LUMO energies (~−2.73 eV) are too high to allow efficient electron injection from either Au or Al electrodes.

**Figure 11:**
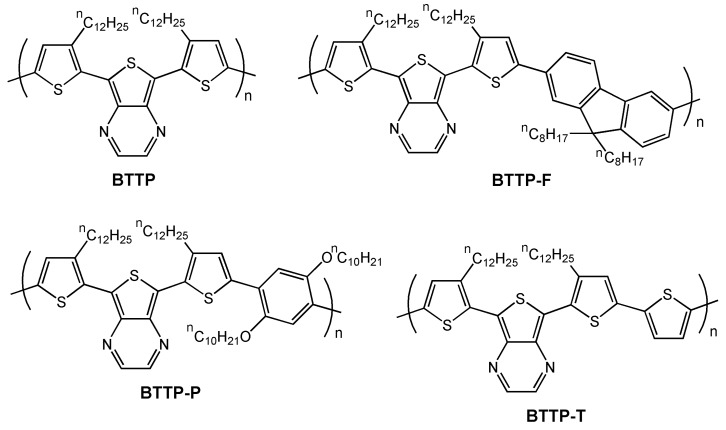
Chemical structures of the semiconducting copolymers **BTTP**, **BTTP-F**, **BTTP-P**, and **BTTP-T**.

In a following paper [66], the same authors synthesized **PTTP**–derived copolymers and tuned the strength of the intramolecular charge transfer by incorporating dioctylfluorene, bis(decyloxy)phenylene or thiophene moieties in the structure (see Figure 11). These copolymers exhibit small optical band gaps in the range 1.1–1.6 eV and ambipolar redox properties. The highest degree of electronic delocalization is found for **BTTP-T**, but this copolymer is poorly soluble.

Bottom-contact devices were fabricated using OTS-functionalized Si/SiO_2_ gate dielectrics, and hole mobilities of 1.1 × 10^-3^ cm^2^V^-1^s^-1^ and 1.6 × 10^-3^ cm^2^V^-1^s^-1^ and on/off ratios of 100 and 2 × 10^4^ were measured for **BTTP-P** and **BTTP-F**, respectively. OFETs based on **BTTP** and **BTTP-T** exhibit lower mobilities of 7.1 × 10^-4^ cm^2^V^-1^s^-1^ and 4.2 × 10^-4^ cm^2^V^-1^s^-1^, respectively. Furthermore, these two copolymers exhibit high “off” currents, very large threshold voltages (>80 V), and low current on/off ratios. Despite these disadvantages, these copolymers have very low band gaps and consequently, broad visible to near infrared absorption, which is especially interesting for photovoltaic cell applications. In a following contribution, the authors focused on the pyrido[3,4-b]pyrazine building block and similar field-effect mobilities, ranging from 4.1 × 10^-4^ to 4.4 × 10^-3^ cm^2^V^-1^s^-1^ were recorded [67].

**Figure 12:**
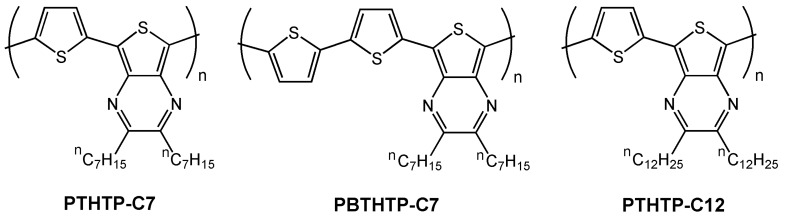
Chemical structures of the semiconducting copolymers **PTHTP-C7**, **PBTHTP-C7**, and **PTHTP-C12**.

Very recently Lee *et al.* [68] reported interesting work using similar molecular units (see Figure 12), in which the effects of donor/acceptor ratio, alkyl side chain length, surface silanization treatment, and thermal annealing on the morphology and charge carrier mobility was investigated. The authors found that lower band gap polymers are achieved when a 1:1 ratio of donor-acceptor groups is used (1.07 eV for **PTHTP-C7** *vs.* 1.38 eV for **PBTHTP-C7**), due to the greater intramolecular charge transfer. Furthermore, the higher molecular weight of **PTHTP-C12** enhances the π-electron delocalization, further decreasing the band gap to 0.97 eV. 

Field-effect transistors fabricated from these polymers on HMDS-treated Si/SiO_2_ substrates exhibited only p-channel characteristics with mobilities of 3.6 × 10^-5^ cm^2^V^-1^s^-1^, 1.2 × 10^-4^ cm^2^V^-1^s^-1^, and 1.1 × 10^-3^ cm^2^V^-1^s^-1^ and current on/off ratios of 3, 300 and 70 for **PTHTP-C7**, **PBTHTP-C7** and **PTHTP-C12**, respectively. The mobility trend the authors reported, **PTHTP-C12 > PBTHTP-C7** >**PTHTP-C7,** differs from the trend found in band gaps and in electron delocalization (**PTHTP-C12 > PTHTP-C7** > **PBTHTP-C7)**, indicating that polymer morphology contributes significantly to the FET performance in addition to the π-delocalization. In order to analyze these effects, the authors used two different approaches on **PTHTP-C12** devices, OTS-8 surface functionalization and thermal treatment. By surface modification, they found an increase of electrical performance on OTS-8-modified surfaces (6.1 × 10^-3^ cm^2^V^-1^s^-1^) due to morphology differences from fibrillar structures on HMDS to a more efficient charge transport nodule-like morphology on OTS treated substrates. Furthermore, by changing the film thermal annealing temperature they were able to increase the hole mobility to 1.1 × 10^-2^ cm^2^V^-1^s^-1^ and to enhance the current on/off ratio (227), due to achieving of a more densely packed grain morphology after removing all traces of solvent. This study points out that device microstructure and parameter optimization can be as important as the search for new molecular/macromolecular structures.

**Figure 13:**
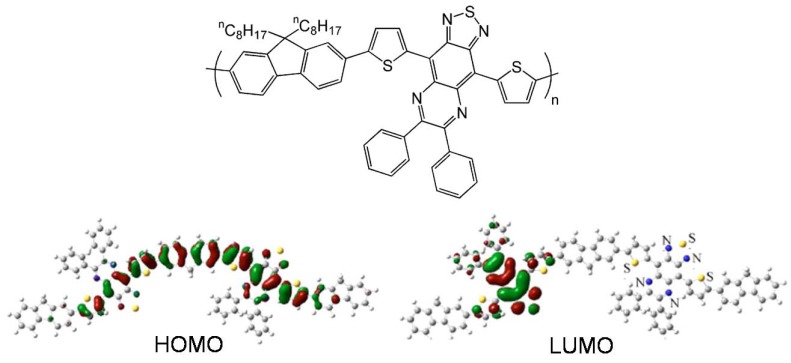
Chemical structure and frontier molecular orbitals topologies of semiconducting copolymer **AFPO-Green1**. Reproduced with permission from reference 69. Copyright 2005 American Institute of Physics.

Another successful strategy to decrease the band gap and enhance FET performance has been demonstrated by Bergreen *et al.* [69], by alternating fluorene and electron donor-acceptor-donor units composed of two electron-rich thiophene rings attached to both sides of a thiadiazolo-quinoxaline electron acceptor group (see chemical structure in Figure 13). The dialkyl fluorene unit was introduced to enhance stability and solubility. This copolymer exhibits a band gap as low as 1.21 eV and a hole mobility in bottom-contact transistors of 0.003 cm^2^V^-1^s^-1^ for a low molecular weight polymer [70] and a value as high as 0.03 cm^2^V^-1^s^-1^ for a higher molecular weight polymer. In this case, the absence of electron transport was explained by the authors on the basis of: (i) the presence of electron trap centers originating from chemical groups on the dielectric surface (unmodified Si/SiO_2_ was used as the gate dielectric); (ii) the localization of the lowest unoccupied molecular orbital (LUMO) of the polymer on the electron-accepting part of the molecule (Figure 13). In contrast, the high hole mobility could be attributed to the delocalized character of the HOMO through the entire conjugated skeleton (Figure 13). The combination of excellent electrical performance with the low band gap makes this copolymer an intringuing candidate for photovoltaic applications.

**Figure 14:**
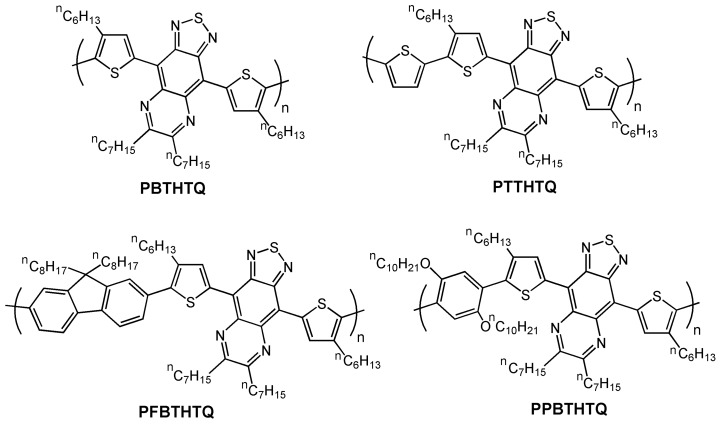
Chemical structures of the semiconducting copolymers **PBTHTQ**, **PTTHTQ**, **PFBTHTQ**, and **PPBTHTQ**.

Following the same donor-acceptor-donor thiophene-thiadiazolo-quinoxaline-thiophene structure motif, a new series of copolymers was recently synthesized and investigated by Chen *et al.* [71]. In this case, various donor groups such thiophene, fluorene, and bis(decyloxy)phenylene were incorporated (see chemical structures in Figure 14). All of the copolymers in that report [71] exhibit low optical band gaps, ranging from 1.05–1.43 eV, due to intramolecular charge transfer (ICT) between the donor and acceptor moieties. Using cyclic voltammetry the authors estimated both the HOMO and LUMO energy levels, and found that incorporating the thiadiazolo quinoxaline moiety greatly increases the electron affinity of the copolymers, lowering the LUMO levels to −3.75 eV in the case of **PBTHTQ**, and ~−3.63 eV for the other polymers. These values are lower than the corresponding one (−3.4 eV) recorded for the ambipolar semiconductor **P(ThdzTh)** [38]. However, bottom-contact devices using OTS-8-treated Si/SiO_2_ exhibit only p-channel characteristics (**PPBTHTQ** was inactive), but it must be noted that only Au electrodes were used while Yamamoto *et al.* [38] used Al contacts to better match the LUMO energy of **P(ThdzTh)**. 

The hole mobilities of **PBTHTQ-**, **PTTHTQ-**, and **PFBTHTQ**-based devices were 1.6 × 10^-4^, 3.8 × 10^-3^, and 1.5 × 10^-4^ cm^2^V^-1^s^-1^ when using high boiling point solvent such as chlorobenzene. Lower mobilities are found when chloroform is used. Electrochemical results points out that the trend found in the electrical performance is in good agreement with the HOMO energy levels (−5.06, −4.84, and −5.12 eV for **PBTHTQ**, **PTTHTQ**, and **PFBTHTQ**, respectively), which indicate the electron-donor strength of the different donor groups. 

**Figure 15:**
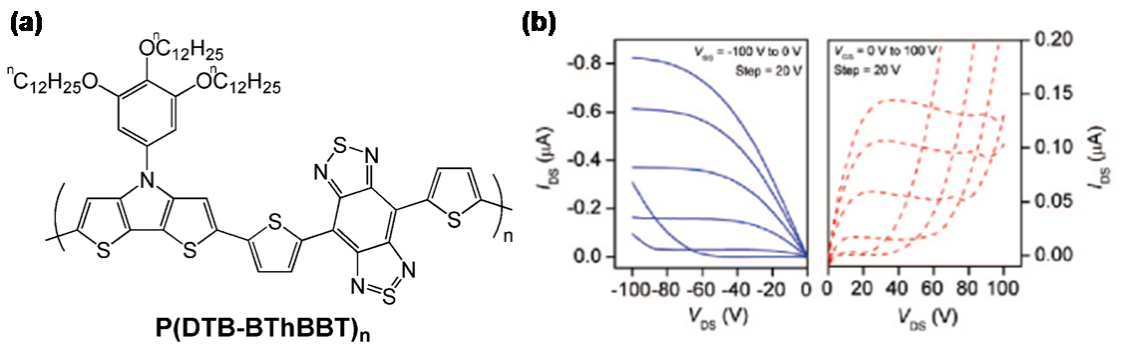
(a) Chemical structure of semiconducting copolymer **P(DTP-BThBBT)_n_**; (b) Output characteristics; p-channel operation (left) is measured in a device annealed at 110 °C; n-channel operation (right) is measured in a device annealed at 150 °C. Reproduced with permission from reference [72]. Copyright 2009 American Chemical Society.

Quite recently, Reynolds *et al.* [72] have reported a low band-gap ambipolar conjugated polymer consisting of the strong donor dithieno[3,2-*b*:2´,3´-*d*]pyrrole functionalized with a trialkoxyphenyl group and the strong acceptor based on benzo[1,2-*c*;4,5-*c´*]bis[1,2,5]thiadiazole (Figure 15a). The authors found a low electrochemical bandgap of only 0.60–0.95 V, ionization potentials of ca. 5.0–5.1 eV and electron affinities of ca. 4.4–4.2 eV, which suggest possible ambipolar charge transport. Field- effect transistors were fabricated in a top-contact geometry using a 200 nm thick SiO_2_ dielectric layer functionalized with either an OTS self-assembled monolayer or a thin buffer layer of benzocyclobutene. The best results were obtained for OTS-treated substrates, where p- (1.2 × 10^-3^ cm^2^V^-1^s^-1^) and n-channel (5.8 × 10^-4^ cm^2^V^-1^s^-1^) electrical characteristics were found for samples annealed at 110 and 150 °C, respectively.

**Figure 16:**
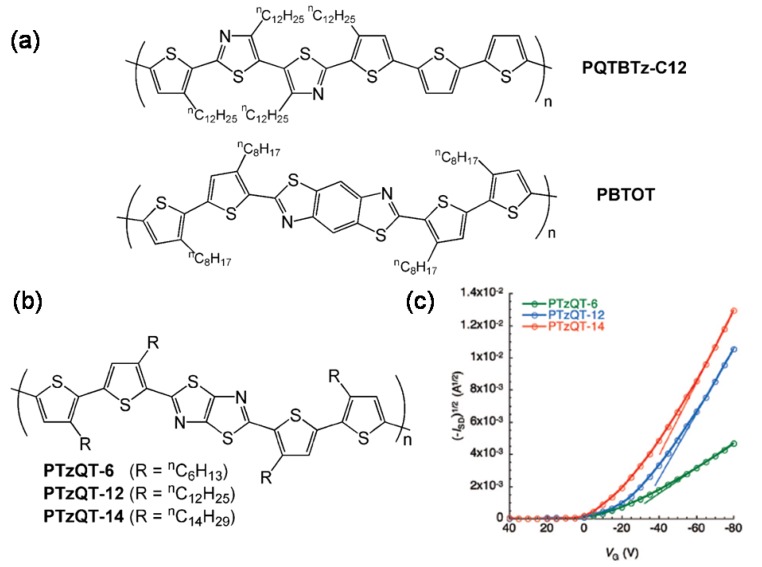
(a) Chemical structure of copolymers **PQTBTz-C12** and **PBTOT**; (b) Chemical structure of copolymers **PTzQT-R**; (c) Evolution of the transfer characteristics of the annealed polymers (150 °C) with alkyl chain length. Reproduced with permission from reference [76]. Copyright 2009 American Chemical Society.

In 2009, Jenekhe *et al.* [73] synthesized a new benzobisthiazole-thiophene copolymer (**PBTOT** in Figure 16a) which, due to its high crystallinity (promoted by the benzobisthiazole unit, as shown in previous sections), exhibited a field-effect mobility of up to 0.01 cm^2^V^-1^s^-1^ and 0.002 cm^2^V^-1^s^-1^ for high and low molecular weight copolymers, respectively, and showed remarkable air stability. Furthermore, preliminary studies on bulk heterojunction solar cells based on the blends of the low molecular weight **PBTOT** and **PC71BM** indicated a power conversion efficiency of 2.1%.

The same year, Bao *et al.* [74] reported a high-performance liquid crystalline semiconducting copolymer, poly(didodecylquaterthiophene-alt-didodecylbithiazole) **PQTBTz-C12** (Figure 16a), which reaches a field-effect mobility as high as 0.33 cm^2^V^-1^s^-1^ when annealed at the mesophase temperature (180 °C). In this copolymer, the unsubstituted bithiophenes units enhance intermolecular π-π stacking while the liquid-crystalline nature results in highly crystalline thin films, which explains the excellent electrical performance. Furthermore, this copolymer exhibits unprecedented bias-stressed stability, comparable to that of a-Si.

Finally, McCullough *et al.* have achieved very good FET performance by combining thiophene and thiazolothiazole building blocks [75,76]. Introduction of the rigid thiazolothiazole unit not only enhances core rigidity, but also increases the conjugation and the ionization potential. Thiophene units with different alkyl chains were analyzed (Figure 16b and Figure 16c) and field-effect mobilities ranging from 0.013–0.30 cm^2^V^-1^s^-1^ were obtained, depending on the alkyl chain and FET channel length used. In particular, the authors found an increase in electrical performance and air stability with longer alkyl chains and ascribed it to the lamellar ordering of the thin films. In these polymers, it is interesting that despite the uneven placement of the alkyl side chains along the backbone, which may suppress interdigitation and crystallinity, very good electrical performance is achieved by the introduction of the fused thiazolothiazole ring.

Table 3 summarizes the electrical properties of the low band gap azine- and azole-derived copolymers presented in this section of the review.

**Table 3:** Summary of the electrical performance (mobility, μ, current on/off ratio, I_on_/I_off_ and threshold voltage, V_T_) of the of various azine- and azole-derived semiconducting copolymers. Hole mobilities and bottom-contact device architectures are utilized unless otherwise specified.

Polymer	Dielectric^a^	μ (cm^2^V^-1^s^-1^)^b^	V_T_ (V)	I_on_/I_off_	Ref.
**Copoly-Non^c^**	H	2.5 × 10^-3^	16	200	58
**P(ThdzTh)^c^**	H	5.4 × 10^-3^ (e.)	−13	3 × 10^4^	38
	H	3.4 × 10^-4^	NR	1 × 10^4^	38
**P(PydTh)-6^c^**	S	3 × 10^-3^	NR	4 × 10^3^	36
**PTHQx**	O-8	3.6 × 10^-3^	−2	6 × 10^5^	64
**BTTP**	O	7.1 × 10^-4^	>80	NR	66
**BTTP-F**	O	1.6 × 10^-3^	−3	2 × 10^4^	66
**BTTP-P**	O	1.1 × 10^-3^	NR	100	66
**BTTP-T**	O	4.2 × 10^-4^	>80	NR	66
**PTHTP-C7**	H	3.6 × 10^-5^	NR	3	68
**PBTHTP-C7**	H	1.2 × 10^-4^	NR	300	68
**PTHTP-C12**	O-8	0.011	21	227	68
**AFPO-Green1**	S	0.03	~0	5 × 10^4^	69
**PBTHTQ**	O-8	1.6 × 10^-4^	NR	215	71
**PTTHTQ**	O-8	3.8 × 10^-3^	NR	505	71
**PFBTHTQ**	O-8	1.5 × 10^-4^	NR	324	71
**PBTOT**	S	0.01	−5.2	>10^6^	73
**PQTBTz-C12**	O-m	0.33	NR	10^7^	74
**PTzQT-14**	O-8	0.20–0.30	−23 to −30	10^5–^10^7^	76
**P(DTB-BThBBT)_n_^c^**	O	1.2 × 10^-3^	NR	NR	72
	O	5.8 × 10^-4^ (e.)	NR	NR	72

^a^ S: untreated Si/SiO_2_ gate dielectric; H: HMDS-functionalized Si/SiO_2_ gate dielectric; O: Octadecyltrichlorosilane-functionalized Si/SiO_2_ gate dielectric; O-8: Octyl-trichlorosilane- and O-m: Octadecyltrimethoxysilane- functionalized Si/SiO_2_ gate dielectrics. ^b^ Hole mobilities are reported unless electron transport (e.) is specified. ^c^ Top-contact devices. NR: Not reported.

## 4. Conclusions 

The effects of introducing azine and azole rings in oligothiophene-based oligomeric and polymeric semiconductors have been summarized in this review, focusing on field-effect transistor applications. Several advantages have been found using this synthetic strategy: (i) The incorporation of nitrogen-containing rings into oligothiophenes and/or polythiophenes lowers the HOMO energy, enhancing their air stability; (ii) The LUMO is also stabilized due to the electron-withdrawing character of these systems, increasing the probability of ambipolar transport; (iii) Due to dipole-dipole interactions, extensive solid state intermolecular π-π interactions are usually found in these donor-acceptor structures, which are desirable to optimize charge transport; (iv) Their electronic and optoelectronic properties can be effectively tuned through intramolecular charge transfer, and (v) In the polymers, the combination of high charge carrier mobilities with very low band gaps makes these structures attractive for photovoltaic applications. However, achieving n-channel charge transport remains challenging, while hole transport is usually slightly depressed compared to oligothiophenes and polythiophenes, due to the insertion of strongly electron-accepting azine- and azole- groups in the chemical structures. 
